# Effect of Electrical Stimulation on Fruit Body Formation in Cultivating Mushrooms

**DOI:** 10.3390/microorganisms2010058

**Published:** 2014-02-12

**Authors:** Koichi Takaki, Kohei Yoshida, Tatsuya Saito, Tomohiro Kusaka, Ryo Yamaguchi, Kyusuke Takahashi, Yuichi Sakamoto

**Affiliations:** 1 Department of Electrical Engineering, Iwate University, 4-3-5 Ueda, Morioka, Iwate 020-8551, Japan; E-Mails: t2313022@iwate-u.ac.jp (K.Y.); t2311027@iwate-u.ac.jp (T.S.); tomohiro.kusaka@toppan.co.jp (T.K.); r-yamaguchi@jreast.co.jp (R.Y.); 2 Morioka Forest Union, 2-9 Konya, Morioka, Iwate 020-0885, Japan; E-Mail: sotoyama@smile.ocn.ne.jp; 3 Iwate Biotechnology Research Center, 22-174-4 Narita, Kitakami, Iwate 024-0003, Japan; E-Mail: sakamoto@ibrc.or.jp

**Keywords:** pulsed power, electrical stimulation, *Lentinula edodes*, mushroom

## Abstract

The effect of high-voltage electrical stimulation on fruit body formation in cultivating mushrooms was evaluated using a compact pulsed power generator designed and based on an inductive energy storage system. An output voltage from 50 to 130 kV with a 100 ns pulse width was used as the electrical stimulation to determine the optimum amplitude. The pulsed high voltage was applied to a sawdust-based substrate of *Lyophyllum decastes* and natural logs hosting *Lentinula edodes*, *Pholiota nameko*, and *Naematoloma sublateritium*. The experimental results showed that the fruit body formation of mushrooms increased 1.3–2.0 times in terms of the total weight. The accumulated yield of *Lentinula edodes* for four cultivation seasons was improved from 160 to 320 g by applying voltages of 50 or 100 kV. However, the yield was decreased from 320 to 240 g upon increasing the applied voltage from 100 to 130 kV. The yield of the other types of mushrooms showed tendencies similar to those of *Lentinula edodes* when voltage was applied. An optimal voltage was confirmed for efficient fruit body induction. The hypha activity was evaluated by the amount of hydrophobin release, which was mainly observed before the fruit body formation. The hydrophobin release decreased for three hours after stimulation. However, the hydrophobin release from the vegetative hyphae increased 2.3 times one day after the stimulation.

## 1. Introduction

Expansion of the use of electric fields and power during cultivation has been occurring for various agricultural crops, especially in the area of horticulture. Cultivation systems have been improved through the use of high-voltage for greenhouse crops such as tomatoes, lettuce, strawberries, and various flowers [1,2,3,4]. The application of a pulsed high voltage to improve the yield in edible mushroom cultivation has also been attempted by some research groups. The fruiting capacity of shiitake mushroom (*Lentinula edodes*; *L. edodes*) was remarkably promoted by applying a pulsed high voltage to log wood [5,6,7]. This effect was also recognized in *L. edodes* fruiting on a mature sawdust-based substrate [8,9]. The fruit body (sporocarp) yield in the electrically stimulated substrate was observed to be 1.7 times more than that in the spontaneous fruiting substrate control [9]. This effect was also recognized in the sporocarp formation of edible mushrooms: *Grifola frondosa*, *Pholiota nameko* (*P*. *nameko*), *Flammulina velutipes*, *Hypsizygus marmoreus*, *Pleurotus ostreatus*, *P.eryngii*, *P. abalones*, and *Agrocybe cylindraceas* [10,11,12]. Sporocarp yield, *i.e.*, fruit body formation in the electrically stimulated substrate, was observed to be 130%–180% greater than that in the spontaneous fruiting substrate control [10]. The pulsed high-voltage stimulation technique was also applied to ectomycorrhizal fungi, which form associations with some types of wood, such as *Laccaria laccata* and *Tricholoma matsutake* [13,14].

Many types of electrical power supplies have been employed to provide electrical stimulation. A large-scale 1 MV high-voltage impulse generator was used to stimulate *L. edodes* log wood [5]. High-voltage AC was used to stimulate an *L. edodes* sawdust substrate [8]. Inductive energy storage (IES) pulsed power generators have favorable features for mushroom-cultivating applications, e.g., they are compact, cost effective, light, and have high voltage amplification compared with capacitive energy storage generators such as the impulse generator [15,16]. The yield of *L. edodes* fruiting bodies was improved with high-voltage stimulation generated by the IES pulsed power generators [6,7]. The effect of the pulsed voltage stimulation on some other types of mushroom such as *P*. *nameko* and *Lyophyllum decastes* (*L. decastes*) was also confirmed using an IES generator developed for the improvement of mushroom yield [11,12]. As a result of these studies, the total harvested weight from log wood and/or sawdust substrates for mushroom cultivation increased by applying a pulsed voltage as an electrical stimulation. 

The mechanism driving the increase in the mushroom yield from the application of a pulsed voltage is not clear, but researchers have suggested two possible explanations. One is that the mushroom hyphae are ruptured by applying a high voltage. Physical damage to the hypha stimulates fruit body formation in mushrooms [8,10]. The other explanation involves the activation of enzymes. Some enzymes are activated by applying a high voltage, and consequently, mushroom fruit bodies develop abundantly [5]. Some effects of pulsed electrical stimulation were recognized using microscopic observation and chemical analysis. A scanning electron microscope (SEM) observation indicated that the synthesis of crump connections was accelerated with electrical stimulation [5,8]. Some types of enzymes, including laccase and protease, were activated by the electrical stimulation [5].

Here, we investigated the effect of electrical stimulation on fruit body formation in cultivating mushroom for industrial applications. The pulsed high voltage was applied to log wood of *L. edodes*, *P*. *nameko*, and *Naematoloma sublateritium* (*N*. *sublateritium*) to evaluate the stimulation effect. The voltage was also applied to *L. decastes* sawdust substrate. An SEM image of the hyphae was used to clarify the influence of applying a high voltage on the mushroom hyphae. The mechanism of the mushroom yield improvement was also investigated by using the polymerase chain reaction (PCR) technique for the released protein analysis. 

## 2. Materials and Methods

### 2.1. Strains

Four different fruiting types were used in this work. Three fruiting types were hosted on natural logs: *L*. *edodes*, *P*. *nameko*, and *N. sublateritium*. The strains of the fruiting types were Yujiro (Mori Co. Ltd., Kiryu, Japan), Mori#3 (Mori Co. Ltd., ), and Mori#77 (Mori Co. Ltd.). One fruiting type was cultured on a sawdust substrate: *L*. *decastes*, LD1 (Miyagi Co., Sendai, Japan). All strains originated from commercial sources and were cultured on potato dextrose agar in Petri dishes. A mycelial disk was inoculated onto the center of the dish, and the dish was incubated at 25 °C.

### 2.2. Culture Media and Growth Conditions

**Figure 1:**
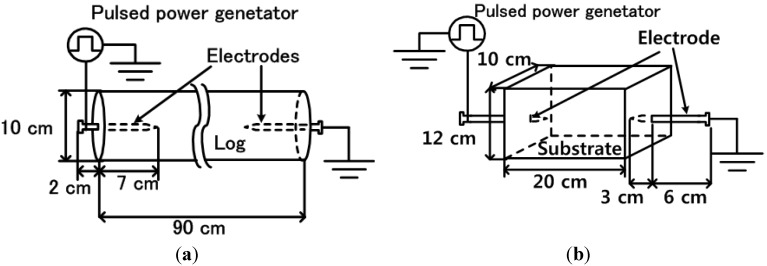
Experimental setup for pulsed voltage stimulation of the bed-log and the sawdust-substrate. (**a**) Bed-log; (**b**) Sawdust block.

The three types of cultivating mushroom—*L*. *edodes*, *P*. *nameko*, and *N. sublateritium*—were inoculated on natural logs of *Quercus crispula Blume* approximately two years before the experiment. The log dimensions were 90 cm in length and 10 cm in diameter, as shown in Figure 1. The number of inoculation points was 35, 30, and 25 for *L*. *edodes*, *P*. *nameko*, and *N. sublateritium*, respectively. The ambient temperature at the inoculation was 5.6 degrees Celsius. The logs were covered with a blackout curtain to maintain the moisture content in the logs hosting the mushroom hyphae. 

The substrate for *L. decastes* cultivation consisted of sawdust from *Quercus serrata* (*Thunb.*) *et Zucc*. supplemented with wheat bran and corn powder produced by Mizudori Co. Ltd. (Kurihara, Japan). The dimensions of the sawdust substrate were 10 cm × 12 cm × 20 cm, and it had a cuboid-block shape. The weight of the substrate was 2.5 kg. The blocks were inoculated with *L. decastes* fungus approximately one year before the stimulation. After inoculation with *L. decastes* fungus, they were matured in a dark room under wet conditions for the necessary period for each experiment. The experiment was carried out in the autumn of 2007 as an attempt to increase the yields of *L. decaste*. The three substrates were used in the groups receiving an applied voltage. The pulsed voltage was applied to a needle electrode with a 3 mm diameter driven into the sawdust block to a depth of 3 cm, as shown in Figure 1b. Three blocks from the control group did not have a pulsed voltage applied. The fruit bodies of *L. decaste* mushrooms were cropped each day.

**Figure 2:**
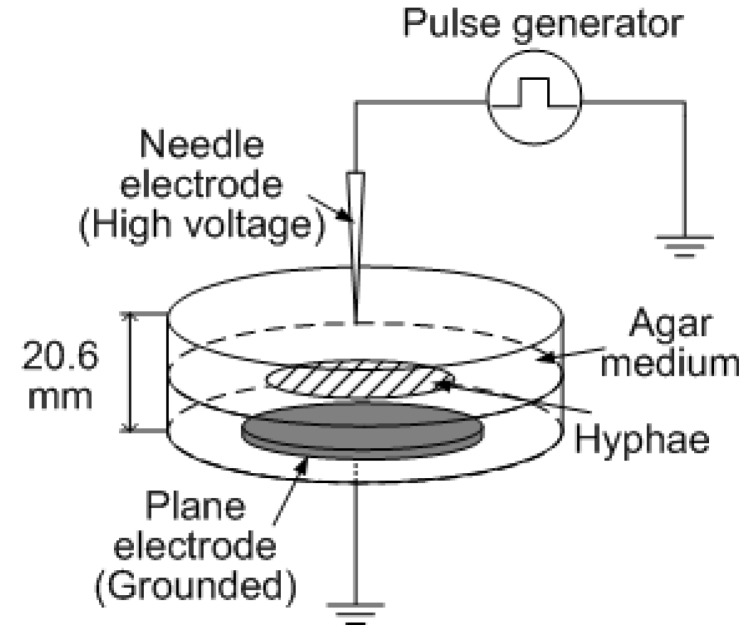
Experimental setup for investigating the effect of pulsed voltage stimulation on the hypha activity.

Figure 2 shows the experimental setup for the pulsed voltage stimulation of an agar medium cultivation of *L. edodes* hypha. *L. edodes.* Hokken No. 600 was used as a specimen. Fungi were inoculated in the center of the agar medium in Petri dishes 5 days before the electrical stimulation. The Petri dish dimensions were 2 cm in depth and 10 cm in diameter. The needle electrode approximately 1 mm in diameter was located in the center of the cover of the Petri dishes, and subsequently, the pulsed voltages were applied by a Blumlein-line type pulsed power generator [17] to provide electrical stimulation. The applied voltage was changed from 20 to 100 kV in amplitude and 100 ns in pulse width. The number of pulse stimulations was fixed at 100. The real-time polymerase chain reaction technique was used to amplify the DNA of the hydrophobin Hyd2 and to measure Hyd2 release [18] using PCR (Applied Biosystems 7500, Applied Biosystems, Foster City, CA, USA).

### 2.3. Electrical Stimulation

Figure 1 shows the experimental setup for the pulsed voltage stimulation of the bed-log and sawdust substrate cultivation systems. The pulsed power generator was constructed based on an IES system and was used to generate pulsed high voltages as electrical stimulation for mushrooms. The IES pulsed power generator basically consisted of primary energy storage capacitors, closing switches, a secondary energy storage inductor, and an opening switch [7,11,12]. The four primary energy storage 0.22 µF capacitors were connected in parallel and were charged up using a high-voltage dc power supply (50 kV maximum voltage). The charging voltage of the each primary energy storage capacitor was controlled in the range from 5 to 7 kV. After charging up the capacitor, the closing switch changed the connection of the capacitors from parallel to series. As a result, the voltage was stepped-up in the same manner as in a Marx generator [12]. The output voltage of the primary circuit was compressed and multiplied by the secondary energy storage inductor and the fuse-type opening switch. The output voltage of the pulsed power generator was varied from 50 to 130 kV with a 100 ns pulse width to determine the optimum amplitude. A needle electrode 3 mm in diameter and 9 cm in length was driven into the logs to a depth of 7 cm, and subsequently, a pulsed voltage was applied by the IES pulsed power generator as a form of electrical stimulation. The temperature rise of the bed-log and the sawdust block by applying voltage was estimated to be several milli-Kelvins using 5.39 J stored energy and 1.3 J/kg specific heat capacity.

### 2.4. Fruiting Body Yield

The fruiting season of the mushroom species is mainly early autumn, with some fruiting also occurring in spring. The experiment was carried out from 2007 to 2008 as an attempt to increase the yields. After the inoculation, the fungi matured on the logs under environmentally controlled conditions. The fruit bodies of mushrooms were cropped every day, and their weight was measured to obtain the yield. Twenty sawdust substrate beds were grouped under the same experimental conditions to obtain as averaged value. Fifteen bed-logs of *L. edodes* were also grouped under the same conditions to study the statistical distribution. The voltage amplitude for the bed-logs was changed to obtain an optimum amplitude for improving mushroom yield, as mentioned in Section 2.3.

## 3. Results

### 3.1. Electrical Characteristics of the Cultivation Bed

**Figure 3:**
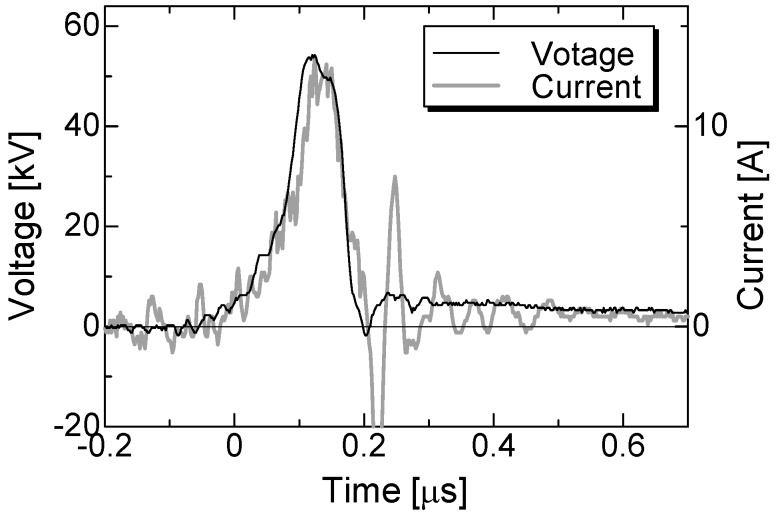
Typical waveforms of the applied voltage and the current through the bed-log at 50 kV applied voltage.

Figure 3 shows typical waveforms of the applied voltage and the current through the bed-log at a 50 kV applied voltage. The waveform of the applied voltage shows a shape typical of IES output and a 100 ns pulse width at full-width half-maximum (FWHM) [12]. The amplitudes of the voltage and the current are 52 kV and 13 A, respectively. The waveform shape of the current is almost in agreement with that of the voltage. This result indicates that the ratio of the voltage to the current, *i.e.*, the resistance between the electrodes, is almost constant while applying the voltage and is approximately 4.0 kΩ. The ratio of 4.0 kΩ almost agrees with the impedance obtained with a multi-meter. The current flows through the sawdust bed uniformly under the experimental conditions because the skin depth is roughly calculated to be 2.1 m [19].

**Figure 4:**
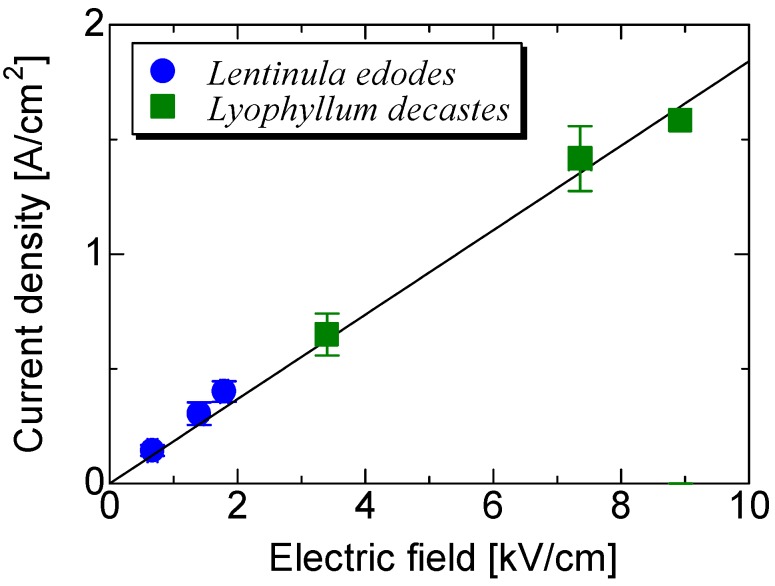
Peak current density in the bed-log and substrate as a function of the applied electric field.

Figure 4 shows the relationship between the current densities in the bed-log or in the substrate and the applied electric field. The current density was obtained by dividing the peak current by cross sections of 78.5 or 120 cm^2^ for the bed-log or the substrate, respectively. The electric field was obtained by dividing the applied voltage by the gap-length of 76 or 14 cm for the bed-log or the substrate, respectively. The electric field and the current density of the substrate are larger than those of the bed-log because the volume of the substrate is smaller than that of the bed-log. The total impedance of the substrate was obtained as 680 Ω by dividing the voltage by the current. The current density increases with a constant gradient as a function of the applied electric field. This result indicates that the conductivity of the substrate almost corresponds to that of the bed-log and is 0.184 S/cm based on the gradient of the slope. 

### 3.2. Fruiting Body Yield in Log Cultivation

#### 3.2.1. Yield of *L. edodes*

**Figure 5:**
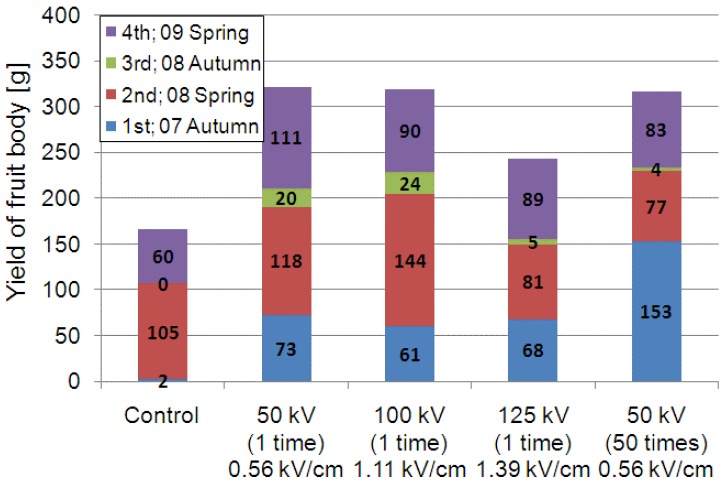
Total weight of cultured *L. edodes* for various electrical stimulation conditions. The total yield are 167, 322, 319, 243, 317 g for control, 50 kV-1 time, 100 kV-1 time, 125 kV-1 time, 50 kV-50 times, respectively.

Figure 5 shows the *L. edodes* crop harvested under five different pulsed voltage stimulation conditions. One group was cultured without pulsed voltage stimulation and was used as a reference, i.e., the control group. Three groups were stimulated by a single high-voltage pulse (one-pulse stimulation) with three different amplitudes: 50, 90, and 125 kV. The last group was stimulated 50 times with a 50 kV pulsed voltage. The yield of the fruit body was evaluated as the total weight harvested during four seasons. It includes the crops from all 15 logs, appropriately averaged without statistical analysis. The yield of the control group was only 2 g in the first harvesting season, autumn of 2007, because the *L. edodes* species used in the present experiment mainly fruits in the spring. In this case, the 30 g weight of fruit bodies was harvested from only one log. Therefore, the standard deviation was 7.5 g, which is larger than the 2 g average weight. This result indicates that the mushroom species employed in the experiment usually does not develop fruit bodies, based on the statistical analysis. However, the yield from the first season increased from 2 to 73 g when a 50 kV pulsed voltage was applied. The yield increased from 73 to 153 g when the number of pulses increased from 1 to 50. In this case, the standard deviation was determined to be 73.0 g, which was lower than the 153 g average weight. This result indicates that the mushrooms developed fruit bodies as the result of applying the pulsed voltages. 

The total harvested weight over four seasons was 167 g in the control group. This value increased to 322 and 319 g when pulsed voltages of 50 and 100 kV were applied, respectively. However, the yield decreased to 243 g when the voltage was increased to 125 kV. This result indicates that an optimum amplitude of the pulsed voltage used for the stimulation of *L. edodes* exists and that it is estimated to range from 50 to 100 kV/m. The number of fruit bodies harvested in 2008 was 46, 48, 36, 40, and 25 in the control, 50 kV × 1, 100 kV × 1, 125 kV × 1, and 50 kV × 50 times groups, respectively. The average weight of one mushroom was 34.2, 43.1, 70.0, 32.3, and 48.6 g. This was calculated using the total crop weight of 1575, 2070, 2520, 1290, and 1215 g. Therefore, the dominant effect of the electrical stimulation used to improve the total mushroom yield in the spring was an increase in the size of each fruit body.

**Figure 6:**
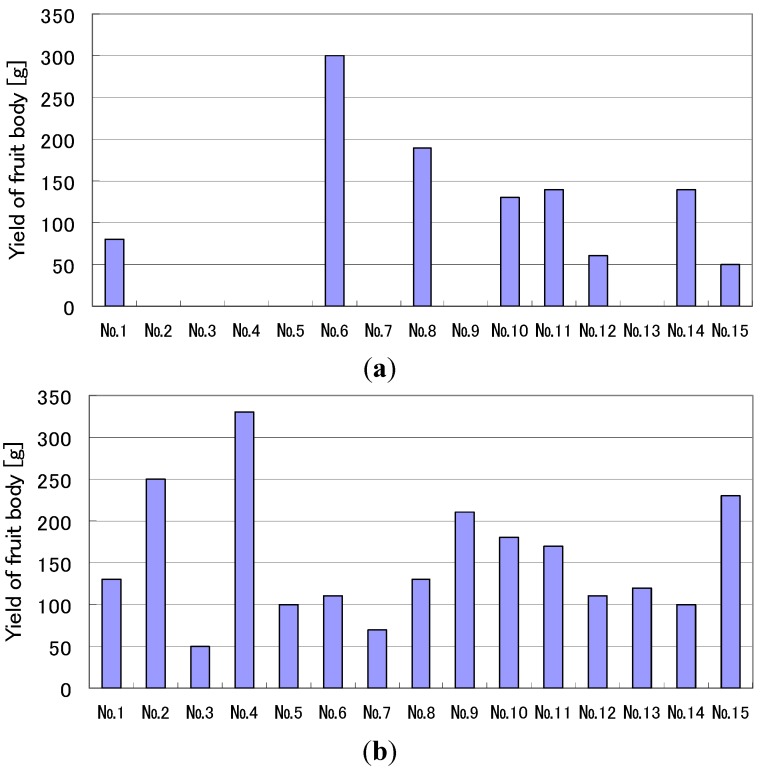
Difference in the yield of fruit bodies of *L. edodes* based on the number of 50 kV applied voltage treatments received. No.1–No.15 indicate labels for each cultivation log. (**a**) one-pulse stimulation; (**b**) 50-pulse stimulation.

Figure 6 shows the weights of *L. edodes* harvested from each log at two different numbers of pulse voltage stimulation. The applied voltage was 50 kV in all cases. The total weight from the logs after 50-pulse stimulation was 2.29 kg (=153 g × 15), as shown in Figure 5, which is larger than the 1.09 kg (=73 g × 15) harvested after a one-pulse stimulation. The maximum value of the harvested fruit body from one log after a one-pulse stimulation was 300 g, which is similar to the 320 g obtained after 50-pulse stimulations. Although there were no logs observed without fruit body formation for 50-pulse stimulation, after a one-pulse stimulation, seven logs contained no fruit bodies. The average yield for one log was approximately 73 g (=1090/15) after a one-pulse stimulation. Only six logs showed a yield larger than the 73 g average value, whereas fourteen logs showed a yield larger than 73 g in the case of 50-pulse stimulation. This result indicates that on particular logs, use of the pulsed voltage decreased the deviation in the mushroom formation. The standard deviations are 27 and 19 g at one- and 50-pulse stimulations, respectively.

#### 3.2.2. Time-History of *L. edodes* Yield

**Figure 7:**
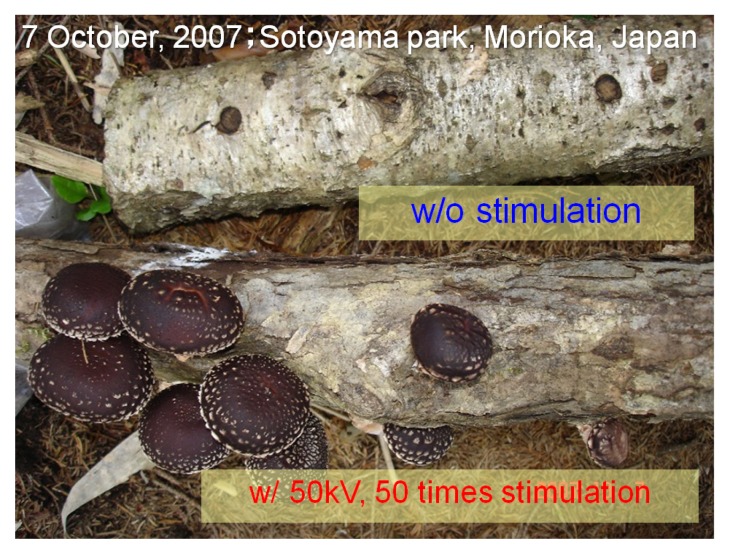
Typical photograph of the cultured *L. edodes* with (**bottom**) and without (**top**) electrical stimulation.

**Figure 8:**
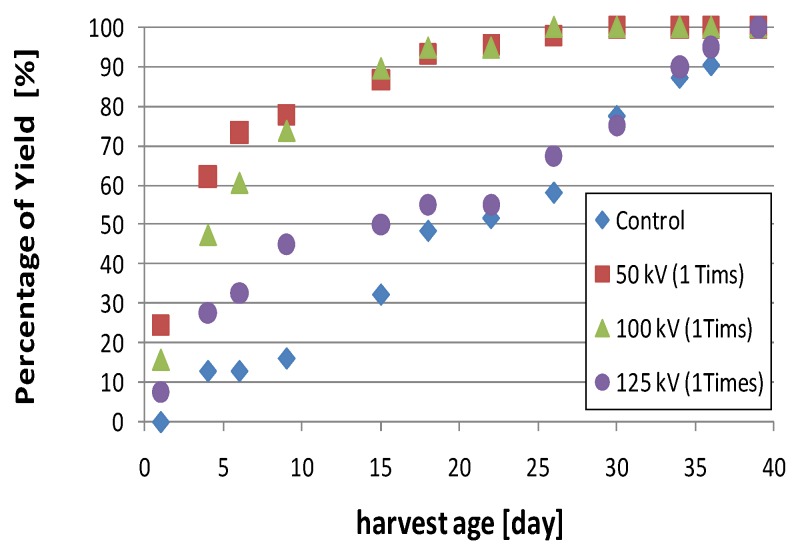
Time-history of the total amount of harvested fruit bodies for various stimulation voltages.

Figure 7 shows a photograph of cultured *L. edodes* taken on the same day. The upper bed-log was used in cultivation without applying a high voltage. The lower bed-log was used in cultivation and a 50 kV voltage was applied 50 times as stimulation. *L. edodes* in the stimulated log grew faster than that in the bed-log without stimulation. The high-voltage electrode is located on the left side of the log. The fruit bodies mainly grow near the high-voltage electrode. Figure 8 shows the history of the amount of mushrooms cultured under various stimulation conditions in the spring of 2009. All the logs were checked every morning, and the growing fruit bodies were harvested throughout the entire harvesting season. The number and the weight of the fruit body harvested from the logs were measured for a period of 40 days. The yield was normalized by the total crop weight for one harvesting season and was evaluated as an aggregate of all crops. The total crop weights were 60, 111, 90, and 89 g in the control, 50, 100, and 125 kV stimulation groups, respectively, as shown in Figure 5. Compared with the control group, the total yield increased when applying a voltage of 50 and 100 kV. The harvested weight for 15 days after the first crop (day 18) was approximately 50% of the total in the control group. However, the crop weight during this period increased to 86% of the total when applying voltages of 50 and 100 kV. This result indicates that the mushrooms can be harvested in fewer days by applying pulsed voltage stimulation.

#### 3.2.3. Yield of *P. nameko* and *N. sublateritium*

**Figure 9:**
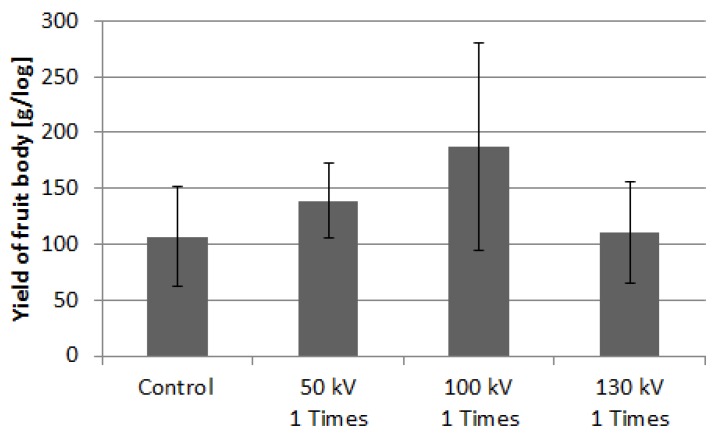
Yield of *Pholiota nameko* fruit bodies for various stimulation conditions. Vertical bars indicate the standard errors of the mean (Number of samples; *n* = 10).

Figure 9 shows the crop weight of *P. nameko* harvested at three different stimulation voltages. One group was cultured without pulsed voltage stimulation and was used as the control group. Three groups were stimulated by a single high-voltage pulse with three different amplitudes; 50, 90, and 130 kV. The yield of the fruit body at the first flash in bed-log cultivation was used for the evaluation. The yield was obtained by dividing the total weight of the crops from 10 bed-logs by the number of logs. The average yield of the control group was approximately 107 (±45) g/log. The average yield increased to 139 (±33) and 187 (±93) g/log by applying a voltage of 50 and 100 kV, respectively. These yields are 1.3 and 1.7 times larger than that of the control group by the evaluation using averaged value. However, these increments are not significant statistically because the standard deviations have large value. The yield decreased to 111 (±46) g when the voltage was increased further, which is almost the same value as that of the control group.

**Figure 10:**
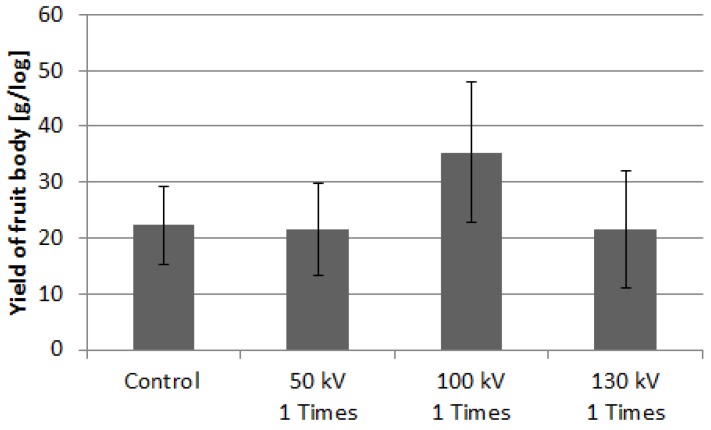
Yield of *Hypholoma sublateritium* fruit bodies for various stimulation conditions. Vertical bars indicate the standard errors of the mean (*n* = 13).

Figure 10 shows the crop weight of *N. sublateritium* stimulated with three different voltage amplitudes; 50, 100, and 130 kV. The yield of the fruit body at the first flash in bed-log cultivation was also used. The average yield was obtained using the total weight harvested from 13 bed-logs. The average yield of the control group was approximately 22.3 (±6.9) g/log. The average yield increased to 35.4 (±12.4) g/log by applying a voltage of 100 kV. It was not statistically significant, but the yield was 1.6 times larger than that of the control group. The applied voltage of 100 kV corresponds to 1.32 kV/cm in an averaged electric field, as shown in Figure 3. This 1.32 kV/cm electric field is similar to 1.1 kV/cm, which is effective as a form of stimulation for fruit body formation, as reported by Tsukamoto based on an evaluation of bed-log cultivation of *L. edodes* [6].

### 3.3. Fruiting Body Yield in Sawdust Substrate Cultivation

**Figure 11:**
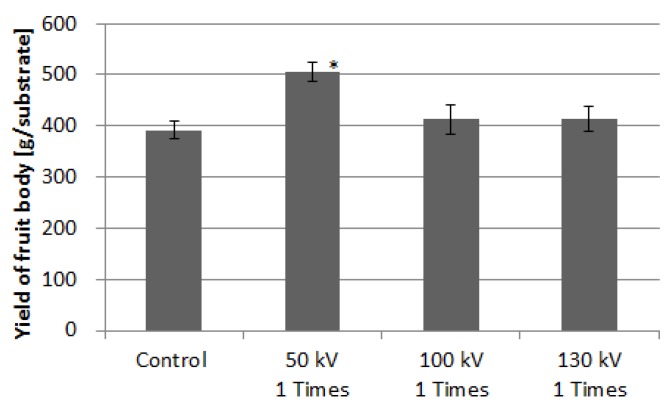
Yield of *Lyophyllum decastes* fruit bodies for various stimulation conditions. Vertical bars indicate the standard errors of the mean (Number of samples; *n* = 20). Asterisks indicate the significant differences at *p* < 0.05 (*).

**Figure 12:**
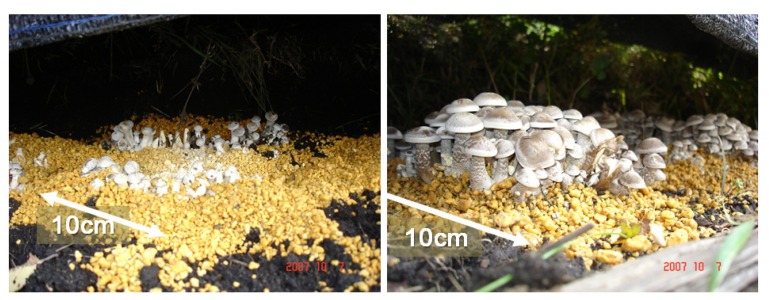
Typical photographs of the cultured *Lyophyllum decastes* without (**left**) and with (**right**) electrical stimulation.

Figure 11 shows the crop weight of *L. decaste* stimulated with three different voltage amplitudes: 50, 90, and 130 kV. The yield of the fruit body at the first flash in substrate cultivation was used. The average yield was obtained using the total weight harvested from 20 substrate beds. The average yield of the control group is approximately 392 (±17) g/substrate. The average yield increased to 505 (±19) g/substrate by applying a voltage of 50 kV. The yield was 1.3 times larger than that of the control group with statistical significance of *p* < 0.05. The applied voltage of 100 kV corresponds to 3.57 kV/cm in an averaged electric field, as shown in Figure 4. Figure 12 shows photographs of cultured *L. decastes* taken the same day. The *L. decastes* in the stimulation group grew faster than those in the control group. This tendency is similar to the experimental result reported by Tsukamoto *et al*. [20]. 

## 4. Discussion

The experimental results indicate that the application of a high voltage to a mushroom bed-log and/or a substrate induces fruit body formation of the mushroom, as shown in Figure 8. The high-voltage application also works as stimulation for fruit body formation, as shown in Figure 6. The effective amplitudes of the voltage for the stimulation are 1.32 and 3.57 kV/cm for the bed-log and the substrate, respectively, as shown in Figure 4, Figure 5, Figure 6, Figure 7, Figure 8, Figure 9, Figure 10 and Figure 11. The results reveal that the mushroom hyphae are activated by applying the voltage. The hypha activity was evaluated by the amount of hydrophobin release, which was mainly observed before the fruit body formation [21].

**Figure 13:**
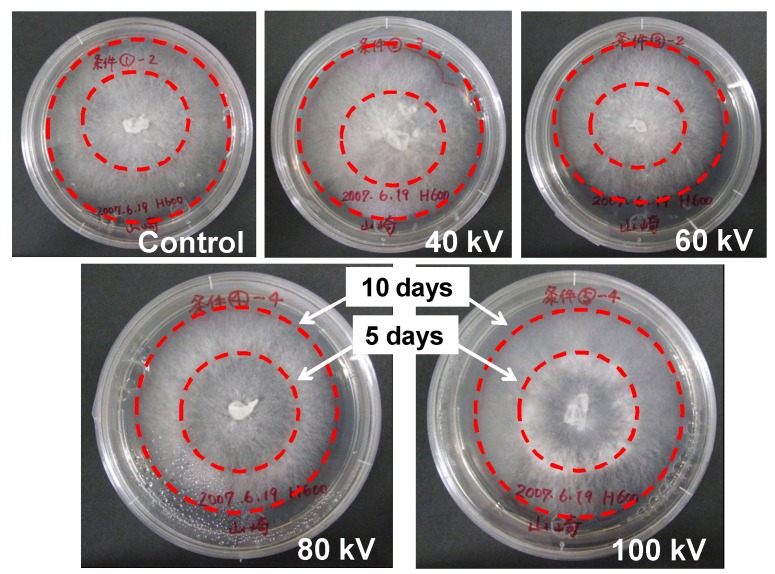
Influence of the pulsed voltage stimulation on hypha growth in agar medium cultivation. The diameter of the Petri dishes is 10 cm in the all cases. The inner and outer dotted circles indicate growth positions of hyphae at 5- and 10-days cultivation, respectively.

Figure 13 shows typical photographs 10 days after cultivation at various amplitudes of the applied voltage. The pulsed voltage was applied after five days of cultivation of *L. edodes* hyphae. The tip positions of the hyphae after 5 days of cultivation were marked by the inner dotted circles. The hyphae grew from the inner to the outer circle positions after 5 days cultivation from the pulse voltage stimulation. From the microscopic observation, the growth direction of the hyphae changed to perpendicular to the surface of the agar medium between the inner and the outer dotted circles as the result of applying a high voltage. 

**Figure 14:**
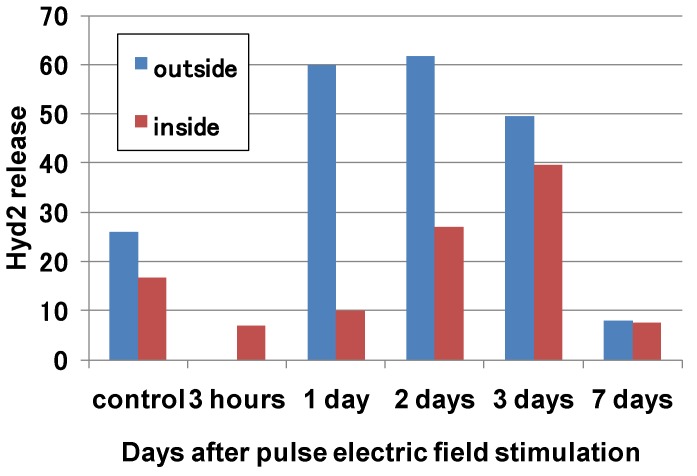
Hydrophobin release for various periods after 100 kV pulsed high-voltage stimulation at two different parts of the hypha. The label “outside” indicates the region between the inner and the outer dotted circles shown in Figure 13. The label “inside” indicates the region inside the inner dotted circles.

Figure 14 shows hydrophobin release from two different parts of the hypha on various days after applying 100 kV pulsed voltage stimulation. The hydrophobin release was analyzed in two parts: vegetative hyphae (outside the inner dotted circles shown in Figure 13) and inactive hyphae (inside the inner dotted circles). The hydrophobin Hyd2 release decreased for three hours after the stimulation. However, the hydrophobin release from the vegetative hyphae increased 2.3 times one day after the stimulation. After that, the hydrophobin Hyd2 release decreased with an increasing number of days after the stimulation. This result indicates that the vegetable hyphae are activated by the pulsed voltage stimulation firstly. The hydrophobin release from the inactive hypha reaches a maximum on the third day after the stimulation. This result indicates that the inactive hyphae are also activated following the vegetable hyphae by the pulsed voltage stimulation. The hyphae of the mushroom can be expressed as a series connection of capacitance and resistance components in same manner to cells, tissue and some microorganisms [22,23]. The applied voltage is divided by the impedance of the capacitance and the resistance components. As the result, a high-frequency component of the electric field can be penetrated inside the cell membrane. A low-frequency of the electric field is mainly applied to the cell membrane [23]. Some researchers reported that the electric fields work as a stimulation for microorganisms such as yeast [24]. The application of a pulsed electric field affects the hyphae via an electrostatic force. This is candidate mechanism that can be used to explain the increase in the hypha activity.

Securing profitability of the electrical stimulation is important for the widespread to the mushroom famers. The pulse voltage stimulation systems for improvement of mushroom yield have been developed and sold by some companies [25]. Typical price of the stimulation system is around five-thousand USD. The increment of *L. edodes* yield is around 155 g/(1-log, 2-year) at 50 kV as shown in Figure 4. The price of the *L. edodes* is around 20 USD/1-kg at natural-log cultivation in Japan [26]. If the mushroom famer uses 1612 logs, the initial cost of 5000 USD can be recovered with increment of the mushroom yield.

## 5. Conclusions

The effect of electrical stimulation on fruit body formation was evaluated using a compact pulsed power generator. A pulsed voltage of 50–130 kV was applied to a sawdust-based substrate of *L. decastes* and natural logs hosting *L. edodes*, *P. nameko*, and *N. sublateritium*. The experimental results showed that the fruit body formation of mushrooms increased 1.3–1.9 times as measured by the total weight. The accumulated yield of *L. edodes* for four cultivation seasons was improved from 160 to 320 g by applying voltages of 50 or 100 kV. However, the yield was decreased from 320 to 240 g when the applied voltage was increased from 100 to 130 kV. The deviation of the mushroom yield among the cultivation logs decreased when applying a pulsed voltage as stimulation for fruit body formation. The yield of the other types of mushrooms showed tendencies similar to those of *L. edodes* as a function of the applied voltage. An optimal voltage was confirmed for efficient fruit body induction. The hypha activity was evaluated by the amount of hydrophobin released, which was mainly observed before the fruit body formation. The hydrophobin release decreased for three hours after stimulation. However, the hydrophobin release from the vegetative hyphae increased to be 2.3 times larger value at one day after the stimulation.
